# Configurational Entropy in Multicomponent Alloys: Matrix Formulation from Ab Initio Based Hamiltonian and Application to the FCC Cr-Fe-Mn-Ni System

**DOI:** 10.3390/e21010068

**Published:** 2019-01-15

**Authors:** Antonio Fernández-Caballero, Mark Fedorov, Jan S. Wróbel, Paul M. Mummery, Duc Nguyen-Manh

**Affiliations:** 1 School of Mechanical Aerospace and Civil Engineering, University of Manchester, Manchester M13 9PL, UK; 2 CCFE, United Kingdom Atomic Energy Authority, Abingdon OX14 3DB, UK; 3 Faculty of Materials Science and Engineering, Warsaw University of Technology, Woloska 141, 02-507 Warsaw, Poland

**Keywords:** multicomponent, ab initio, configuration entropy, matrix formulation, cluster expansion, cluster variation method, monte carlo, thermodynamic integration

## Abstract

Configuration entropy is believed to stabilize disordered solid solution phases in multicomponent systems at elevated temperatures over intermetallic compounds by lowering the Gibbs free energy. Traditionally, the increment of configuration entropy with temperature was computed by time-consuming thermodynamic integration methods. In this work, a new formalism based on a hybrid combination of the Cluster Expansion (CE) Hamiltonian and Monte Carlo simulations is developed to predict the configuration entropy as a function of temperature from multi-body cluster probability in a multi-component system with arbitrary average composition. The multi-body probabilities are worked out by explicit inversion and direct product of a matrix formulation within orthonomal sets of point functions in the clusters obtained from symmetry independent correlation functions. The matrix quantities are determined from semi canonical Monte Carlo simulations with Effective Cluster Interactions (ECIs) derived from Density Functional Theory (DFT) calculations. The formalism is applied to analyze the 4-body cluster probabilities for the quaternary system Cr-Fe-Mn-Ni as a function of temperature and alloy concentration. It is shown that, for two specific compositions (Cr${}_{25}$Fe${}_{25}$Mn${}_{25}$Ni${}_{25}$ and Cr${}_{18}$Fe${}_{27}$Mn${}_{27}$Ni${}_{28}$), the high value of probabilities for Cr-Fe-Fe-Fe and Mn-Mn-Ni-Ni are strongly correlated with the presence of the ordered phases L1${}_{2}$-CrFe${}_{3}$ and L1${}_{0}$-MnNi, respectively. These results are in an excellent agreement with predictions of these ground state structures by ab initio calculations. The general formalism is used to investigate the configuration entropy as a function of temperature and for 285 different alloy compositions. It is found that our matrix formulation of cluster probabilities provides an efficient tool to compute configuration entropy in multi-component alloys in a comparison with the result obtained by the thermodynamic integration method. At high temperatures, it is shown that many-body cluster correlations still play an important role in understanding the configuration entropy before reaching the solid solution limit of high-entroy alloys (HEAs).

## 1. Introduction

Multicomponent systems, called High Entropy Alloys (HEAs), are crystalline solids that form predominantly in a single phase. These systems were brought to wider attention through the work of Cantor et al. [1] and Yeh et al. [2]. At or near equiatomic ratio of compositions, the configuration entropy is maximized and given by the formula $S_{config}/R=lnK$ where *R* is the ideal gas constant, and *K* is the total number of different components. It has been proposed to define K=4 as the minimum number of components at or near equiatomic composition for these systems to be called HEAs [3,4,5].

There are various sources of entropy in a system in addition to configuration entropy including vibration, electronic and magnetic contributions. The co-existence of multiple phases in the equilibrium state of a chemical system with a given overall composition at a specified temperature is found by minimizing the Gibbs free energy of the whole system. When the system undergoes a phase transition of either order-disorder or spinodal decomposition, the relative differences of configurational entropy are dominant in comparison to those contributions from vibration and magnetic terms in FeCoCrNi [6] where the relative difference of configuration entropy dominates or is of similar magnitude to vibration entropy. These multicomponent alloys have transition metals Co, Cr, Fe, and Ni that exhibit magnetic behavior and have magnetic phase transitions characterized by Curie temperatures sensitive to concentrations [7].

In general, the formulation of configuration entropy given by the expression $S_{config}/R=lnK$ only holds for disordered alloys with equiatomic composition at temperatures near their melting point. On the other hand, at low temperatures, the lowest free energy structures can be ordered intermetallic phases or partially ordered structures. In the intermediate temperature range, there exists short range order, which is related to the nature of the chemical environment of each atomic species, containing ordering or segregation preferences specially at low temperatures, because random disordering is favored at the high temperatures. The importance of short-range order (SRO) was manifested in diffuse X-ray scattering measurements of alloys containing ordered superstructure domains. The SRO effect is also seen to significantly affect electrical resistivity properties [8,9], and to influence the alloy strength by hindering dislocation motion [10] in concentrated alloys. Recently, it has been demonstrated how short-range order can be predicted from ab initio based Hamiltonian in combination with Monte Carlo simulations [11] in multi-component Mo-Nb-Ta-V-W systems. The study predicted that a strong SRO parameter may lead to the formation of Mo-Ta ordering in the B2 structure after quenching down from temperatures as high as 3000 K. HEAs such as Al${}_{1.3}$CoCrCuFeNi have been reported to be susceptible to complex phase transitions including segregation, precipitation, chemical ordering and spinodal decomposition into a complex microstructure containing regions of Body-Centered Cubic (BCC), Face-Centered Cubic (FCC) and B2 phases [12]. Physical models have been successfully applied to predict the formation of these phases on the basis of the average number of valence electrons [13].

The prediction of equilibrium thermodynamic properties (free energies, and phase diagrams) is one of the goals of computational materials science. Lattice statistical models involving an Ising-like Hamiltonian developed from ab initio enthalpies of mixing have become an important tool in the computation of the thermodynamic properties of alloy systems. In particular, the cluster expansion method [14,15,16] expands the Hamiltonian into contributions from an optimized set of clusters, each term weighted by Effective Cluster Interactions. The thermodynamic properties at temperature are obtained from the ECIs by computing the free energies from semi-canonical Monte Carlo simulations in combination with the thermodynamic integration technique [17].

The Cluster Variation Method (CVM) formalism [18,19] expresses the free energy in terms of enthalpies of mixing and configuration entropies as a function of the temperature dependence for a specific set of clusters by minimizing the free energy from variational principles. The correlation function parameters in terms of which configuration entropy and enthalpy of mixing are expressed grow exponentially with component number *K* and size of the maximal cluster ω. Due to this, the CVM has been commonly applied to clusters consisting of four sites in a regular tetrahedron or the so-called tetrahedron-octahedron in the FCC lattice [20,21], or the four sites in a irregular tetrahedron in the BCC lattice [22]. Within the tetrahedron approximation, the CVM calculations for the FCC involve the empty lattice, isolated points, first nearest neighbor pair, triangle and tetrahedron [20]. It is generally accepted that the integrated Monte Carlo method is more accurate, but the calculations are time consuming due to the need for many passes to obtain free energies at any given temperature from the disordered high temperature configuration. A hybrid approach taking the Monte Carlo calculated correlation functions at temperature and computing the configuration entropy value from analytic CVM expression has been used in a binary FCC lattice model [23]. It is shown that accurate free energies can be obtained for ordered and disordered phases at arbitrary chemical concentration and temperature without thermodynamic integration provided that use is made of high-order CVM entropy expressions. However, the clusters optimized from the Cluster Expansion (CE) method reported in the literature do not often match those used for the CVM. More importantly, calculations of the configuration entropy for multi-component systems represent a very serious challenge for computational materials science. In this work, we close this gap by developing a new methodology based on matrix formulation to calculate analytically the cluster probabilities for arbitrary *K*-component alloys from the correlation functions obtained by the hybrid Monte Carlo and CE Hamiltonian. We apply our method to the FCC four-component CrFeMnNi system for investigating configuration entropy as a function of temperature and alloy composition. Two specific compositions are chosen to illustrate the importance of many-body cluster probability functions in multi-component systems. The first one is equatomic composition $Cr_{25}Fe_{25}Mn_{25}Ni_{25}$, which is of a great interest for the HEA community. The second one is $Cr_{18}Fe_{27}Mn_{27}Ni_{28}$ which has been used recently to design radiation tolerant materials for advanced nuclear reactor systems. This alloy has been studied in preference to Cantor’s one [1] due to the removal of Co, which can cause activation by transmutation of ${}^{60}$Co isotope in nuclear reactors [24].

This paper is organized as follows. In Section 2, a new matrix formulation for a K-component system based on the orthonormal sets of cluster expansion initially introduced within the Alloy Theoretic Automated Toolkit (ATAT) program [15,16] is presented. This formulation provides a rigorous link of cluster correlation functions obtained from Monte Carlo simulations with multi-body probabilities. Notation is developed to treat as general as possible arbitrary cluster sizes, ω, number of components, *K*, and temperature. In Section 3, the hybrid method is illustrated to calculate 4-body cluster probabilities as a function of temperature for the two specific compositions of equiatomic Cr${}_{25}$Fe${}_{25}$Mn${}_{25}$Ni${}_{25}$ and also the composition Cr${}_{18}$Fe${}_{27}$Mn${}_{27}Ni_{18}$ from [24]. Here, the connection with the tetrahedron approximation in CVM is discussed due to the presence of a first nearest-neighbor 4-body cluster interaction within the investigated CE Hamiltonian. In Section 4, configuration entropy is discussed at two temperatures 1000 and 3000 K for different cluster decorations obtained from the CE method and compared with the integrated Monte Carlo results. The changes in configuration entropy are attributed to the presence of ordered phases that are more stable at low temperatures and the complementary tendency towards disordered random solution of the alloy at the given average composition. The main conclusions of this work are given in Section 5.

## 2. Methods

### 2.1. Matrix Formulation of Cluster Expansion

Let us consider an alloy system with arbitrary number, *K*, of components and crystalline lattice symmetry, in which the disordered phase is described by space group, *G*. The CE Hamiltonian at T=0 K of the alloy can be found from enthalpies of mixing, $\Delta H_{DFT}^{Mixing}\left[\vec{\sigma }\right]$, of derivative alloy structures [16,25] denoted by varying length arrays of spin-like variables ${\vec{\sigma }}_{i}$ taking values from 0 to K−1. In general, a derivative structure of the alloy has non-zero atomic concentrations for each of p=1,…,K different chemical elements forming it i.e., $x{\left[\vec{\sigma }\right]}_{p}\neq 0$. For each member structure, the reference energy in the calculation of the enthalpy of mixing is the pure element total energy, $E_{total}\left[p\right]$, with the same crystallographic symmetry. The alloy enthalpy of mixing is defined as follows:

(1) $$\Delta H_{DFT}^{Mixing}\left[\vec{\sigma }\equiv \left\{\sigma_{1},\sigma_{2},\cdots \right\}\right]=E_{total}\left[\vec{\sigma }\equiv \left\{\sigma_{1},\sigma_{2},\cdots \right\}\right]-\sum_{p=1,\ldots ,K}x_{p}\left[\vec{\sigma }\equiv \left\{\sigma_{1},\sigma_{2},\cdots \right\}\right]E_{total}\left[p\right].$$

The enthalpies of mixing are calculated from the total energies $E_{total}\left[\vec{\sigma }\right]$ and $E_{total}\left[p\right]$ of the alloy and the pure element systems, respectively, by density functional theory for the underlying lattice. In the CE, the $\Delta H_{DFT}^{mixing}\left[\vec{\sigma }\right]$ of an arbitrary structure $\vec{\sigma }$ is expanded into a sum of reference clusters, $\Delta H_{CE}^{Mixing}\left[\vec{\sigma }\right]$ and can be written in the following formula [15,16,17]:

(2) $$\Delta H_{CE}^{Mixing}\left[\vec{\sigma }\equiv \left\{\sigma_{1},\sigma_{2},\cdots \right\}\right]=\sum_{\omega ,n,(s)}m_{\omega ,n}^{\left(s\right)}J_{\omega ,n}^{\left(s\right)}{\langle \Gamma_{\omega^{\prime },n^{\prime }}^{\left(s^{\prime }\right)}\left[\vec{\sigma }\equiv \left\{\sigma_{1},\sigma_{2},\cdots \right\}\right]\rangle }_{\omega ,n,(s)}.$$

In Equation (2), each reference cluster is characterized by three labels ω,n and (s). The label ω denotes the total number sites; *n* is an auxiliary label that refers to highest order coordination shell contained in the reference cluster; and finally the label (s) = ($j_{1},j_{2},\cdots ,j_{\omega }$) denotes decoration of the cluster by point functions with dimension equal to ω and $j_{i}$ taking values 0,…,K−1. For each reference cluster, there is an associated effective cluster interaction $J_{\omega ,n}^{\left(s\right)}$, and multiplicity per lattice site, $m_{\omega ,n}^{\left(s\right)}$. The term ${\langle \Gamma_{\omega^{\prime },n^{\prime }}^{\left(s^{\prime }\right)}\left[\vec{\sigma }\right]\rangle }_{\omega ,n,(s)}$ used in the definition of enthalpy of mixing in Equation (2) is the thermally averaged cluster correlation function over all clusters $\omega^{\prime },n^{\prime },\left(s^{\prime }\right)$ which are equivalent to a space group symmetry element of the disordered phase within the reference decorated cluster defined by ω,n,(s). Overall, the triple product of multiplicity, ECI and correlation function, $m_{\omega ,n}^{\left(s\right)}J_{\omega ,n}^{\left(s\right)}{\langle \Gamma_{\omega^{\prime },n^{\prime }}^{\left(s^{\prime }\right)}\left[\vec{\sigma }\right]\rangle }_{\omega ,n,(s)}$ gives the energetic contribution per lattice site of the reference cluster ω,n and (s) to the enthalpy of mixing of the particular structure configuration given by $\vec{\sigma }$.

For multicomponent systems, the choice of a set of basis functions is important for the matrix formulation of the CE method [14]. The simplest set consists of the successive K−1 powers of the pseudo-spin configuration variable {σ} as was originally suggested by Taggart [26]. In this work, the correlation function is defined as the product of point functions initially proposed in the ATAT program [15,16]. Configurational average of the correlation functions is then given by the following formula:

(3) $$\begin{array}{cc} {\langle \Gamma_{\omega^{\prime },n^{\prime }}^{\left(s^{\prime }\right)}\left[\vec{\sigma }\right]\rangle }_{\omega ,n,(s)} & =\langle \gamma_{j_{1},K}\left[\sigma_{1}\right]\gamma_{j_{2},K}\left[\sigma_{2}\right]\cdots \gamma_{j_{\omega },K}\left[\sigma_{\omega }\right]\rangle \\ & =\frac{1}{\Omega [\omega ,n]}\sum_{u=1}^{\Omega [\omega ,n]}\gamma_{j_{1},K}\left[\sigma_{{\vec{\left(\omega ,n\right)}}_{u_{1}}}\right]\gamma_{j_{2},K}\left[\sigma_{{\vec{\left(\omega ,n\right)}}_{u_{2}}}\right]\cdots \gamma_{j_{\omega },K}\left[\sigma_{{\vec{\left(\omega ,n\right)}}_{u_{\omega }}}\right], \end{array}$$

where the point function is written as:

(4) $$\gamma_{j,K}\left[\sigma_{i}\right]=\left\{\begin{array}{cc} 1, & \;\mathrm{if}\;j=0,\\ -\cos\left(2\pi \left\lceil \frac{j}{2}\right\rceil \frac{\sigma_{i}}{K}\right), & \;\mathrm{if}\;j>0\;\mathrm{and}\;\mathrm{odd},\\ -\sin\left(2\pi \left\lceil \frac{j}{2}\right\rceil \frac{\sigma_{i}}{K}\right), & \;\mathrm{if}\;j>0\;\mathrm{and}\;\mathrm{even}, \end{array}\right.$$

In Equation (3), the correlation function of the system is averaged over the arbitrary crystal structure of the alloy system with configuration $\vec{\sigma }$ over all the set of Ω[ω,n] clusters, ${\left\{\overset{↔}{{\left(\omega ,n\right)}_{u}}\right\}}_{u=1,2,\cdots ,\Omega [\omega ,n]}$ where each cluster labeled by *u* contains ω sites, each site denoted by the vector, ${\vec{\left(\omega ,n\right)}}_{u_{i}}$. The *i*-union of the ω vectors, ${\vec{\left(\omega ,n\right)}}_{u_{i}}$, composes the cluster of vectors, $\left({\vec{\left(\omega ,n\right)}}_{u_{1}},{\vec{\left(\omega ,n\right)}}_{u_{2}},\cdots ,{\vec{\left(\omega ,n\right)}}_{u_{\omega }}\right)\equiv \overset{↔}{{\left(\omega ,n\right)}_{u}}$. The $u_{1},u_{2},\cdots ,u_{\omega }$ are numeric labels referring to the values taken by variable *u* in Equation (3) that serve to enumerate the vectors making up the *u* cluster of vectors $\left\{\overset{↔}{{\left(\omega ,n\right)}_{u}}\right\}$. The clusters ${\overset{↔}{\left(\omega ,n\right)}}_{u}$ are symmetrically equivalent to the reference cluster (ω,n) sites by a symmetry operation and are enumerated by an index taking values 1,2,⋯,Ω[ω,n]. Ω[ω,n] is defined as the number of times the reference cluster (ω,n) is contained in a structural configuration which can be obtained from Monte Carlo simulation. The sites in the cluster $\left\{\overset{↔}{{\left(\omega ,n\right)}_{u}}\right\}$ can allocate all of the possible decoration values ∈(s) which we express as $\left(s\right)=\left(j_{1},j_{2},\cdots ,j_{\omega }\right)$. These integer indexes corresponding to the decorated cluster are parameters for the point functions in Equation (4).

For an arbitrary ω-sites cluster and *K* components, the number of ω-tuples formed with integer entries running from 0⋯K−1 can be calculated. For ω=2, the formula calculates the number of symmetrically unique decorations (s) for a two-point cluster ${\langle \Gamma_{\omega^{\prime },n^{\prime }}^{\left(s^{\prime }\right)}\left[\vec{\sigma }\right]\rangle }_{\omega =2,n,(s)}$ and *K* component alloy system reduces to (K+1)K/2 [11], where *K* is the number of components. For higher order clusters, the total number of decorations depend on the cluster coordinates and the space group symmetry *G* of the high temperature disordered phase i.e., FCC or BCC and can not be simply expressed in terms of K (in general there could be less than ω+K−1K−1=(ω+K−1)!(K−1)!(ω)! number of symmetrically unique correlation functions). ATAT [15,16] numerically works out all the number of symmetrically unique decorated clusters $\omega^{\prime },n^{\prime },\left(s^{\prime }\right)$ equivalent to ω,n,(s) and uses one correlation function per set of equivalent decorated clusters ${\langle \Gamma_{\omega^{\prime },n^{\prime }}^{\left(s^{\prime }\right)}\left[\vec{\sigma }\right]\rangle }_{\omega ,n,(s)}$. For convenience in notation, from now on, we will use $\langle \Gamma_{\omega ,n}^{\left(s\right)}\left[\vec{\sigma }\right]\rangle ={\langle \Gamma_{\omega^{\prime },n^{\prime }}^{\left(s^{\prime }\right)}\left[\vec{\sigma }\right]\rangle }_{\omega ,n,(s)}$. In general, two decorations (s) and $\left(s^{\prime }\right)$ are symmetrically equivalent if there is at least one element in the space group symmetry g∈G that transforms the sites $\left\{\vec{\tau_{1}},\vec{\tau_{2}},\cdots ,\vec{\tau_{\omega }}\right\}\subset \left(\omega ,n\right)$ into $\left\{{\vec{\tau }}_{g[1]},{\vec{\tau }}_{g[2]},\cdots ,{\vec{\tau }}_{g[\omega ]}\right\}\subset \left(\omega ,n\right)$ as the permutation connecting the decorations $\left(s\right)=\left(1,2,\cdots ,\omega \right)\subset \{{\left(s\right)}_{\left(\omega ,n\right)}\}$ and $\left(s^{\prime }\right)=\left(g\left[1\right],g\left[2\right],\cdots g\left[\omega \right]\right)\subset \left\{{\left(s\right)}_{\left(\omega ,n\right)}\right\}$. This is given by the following equation:

(5) $$g\left\{\vec{\tau_{1}},\vec{\tau_{2}},\cdots ,\vec{\tau_{\omega }}\right\}=\left\{{\vec{\tau }}_{g[1]},{\vec{\tau }}_{g[2]},\cdots ,{\vec{\tau }}_{g[\omega ]}\right\}.$$

With the symmetry relations between decorations (s) and $\left(s^{\prime }\right)$ in any given cluster, we are able to retrieve all $K^{\omega }$ possible ω-tuples from the set of unique symmetries corresponding to an ω cluster. The point functions $\gamma_{j,K}\left[\sigma_{i}\right]$ used to define the general correlation functions are related to the multi-body cluster probabilities (see below in Equation (9)) by direct products of a linear transformation [27], $\tau_{K}$

(6) $${\left(\tau_{K}\right)}_{ji}\equiv \gamma_{j,K}\left[\sigma_{i}\right],$$

where the new matrix τ is constructed from these point functions,$\gamma_{j,K}\left[\sigma_{i}\right]$. It can be trivially shown that the inverse of Van der Monde matrices with complex entries equal to roots of unity is its complex conjugate. The inverse of the $\tau_{K}$ matrix can be obtained from the complex conjugate matrix of $\tau_{K}$ by taking the real and imaginary part and riffling their rows. From Equation (4), the following expression results for a system with K components:

(7) $${\left(\tau_{K}^{-1}\right)}_{ij}=\left\{\begin{array}{cc} \frac{1}{K}, & \;\mathrm{if}\;j=0,\\ -\frac{2}{K}\cos\left(2\pi \left\lceil \frac{j}{2}\right\rceil \frac{\sigma_{i}}{K}\right), & \;\mathrm{if}\;j>0\;\mathrm{and}\;j-1<K\;\mathrm{and}\;j\;\mathrm{odd},\\ -\frac{2}{K}\sin\left(2\pi \left\lceil \frac{j}{2}\right\rceil \frac{\sigma_{i}}{K}\right), & \;\mathrm{if}\;j>0\;\mathrm{and}\;j\;\mathrm{even},\\ -\frac{1}{K}\cos\left(2\pi \left\lceil \frac{j}{2}\right\rceil \frac{\sigma_{i}}{K}\right), & \;\mathrm{if}\;j-1=K\;\mathrm{and}\;j\;\mathrm{odd}. \end{array}\right.$$

To the best of our knowledge, Equation (7) represents a new formulation for the inverse of $\tau_{K}$ matrix to ensure that the basis set defined by Equation (4) is rigorously orthonormal. The size of $\tau_{K}$ matrix is *K*x*K*, where *K* is the number of components. It is convenient to perform the matrix multiplications with all $K^{\omega }$ decorations formed from ω-tuples with integer elements running from 0 to K−1. In particular, for the case of four component K=4, these matrices τ become by applying Equations (4) and (7) for the inverse $\tau_{K}^{-1}$:

(8) $$\begin{array}{cc} \tau_{4}=\left(\begin{array}{cccc} 1 & 1 & 1 & 1\\ -1 & 0 & 1 & 0\\ 0 & -1 & 0 & 1\\ -1 & 1 & -1 & 1 \end{array}\right) & \;\;\;\tau_{4}^{-1}=\frac{1}{4}\left(\begin{array}{cccc} 1 & -2 & 0 & -1\\ 1 & 0 & -2 & 1\\ 1 & 2 & 0 & -1\\ 1 & 0 & 2 & 1 \end{array}\right). \end{array}$$

We duplicate the symmetrically unique decorations (s) whenever two decorations are connected by symmetry of the disordered structure. For relating two equivalent decorations, we find it convenient to use a permutation representation of the space group operator as permutations of ω-site tuples. We use the property of invariance of the cluster expansion to obtain probability distributions from correlation functions [28]. As a consequence of the compact formalism and by using Equation (4) and (7), the expression of correlation functions can be rewritten into a matrix form:

(9) $$\langle \Gamma_{\omega ,n}^{\left(ij\cdots \right)}\left[\vec{\sigma }\right]\rangle =\sum_{\forall [(pq\cdots )]}^{K^{\omega }}\gamma_{i,K}\left[{\vec{\sigma }}_{p}\right]\gamma_{j,K}\left[{\vec{\sigma }}_{q}\right]\cdots y_{\omega ,n}^{\left(pq\cdots \right)}\left[\vec{\sigma }\right]\equiv {\overset{\omega }{\overbrace{\left(\tau_{K}^{}⊗\cdots ⊗\tau_{K}^{}\right)}}}_{ij\cdots ,pq\cdots }y_{\omega ,n}^{\left(pq\cdots \right)}\left[\vec{\sigma }\right].$$

With the aid of the matrix formulation from Equation (9) and the generalized form of the inverse matrix $\tau_{K}^{-1}$ in Equation (7), one can express the ω-cluster probabilities into a matrix form:

(10) $$y_{\omega ,n}^{\left(pq\cdots \right)}\left[\vec{\sigma }\right]={\overset{\omega }{\overbrace{\left(\tau_{K}^{-1}⊗\cdots ⊗\tau_{K}^{-1}\right)}}}_{pq\cdots ,ij\cdots }\langle \Gamma_{\omega ,n}^{\left(ij\cdots \right)}\left[\vec{\sigma }\right]\rangle ,$$

where we have used the notation for direct product of matrices ${{\overbrace{\left(\tau_{K}^{-1}⊗\cdots ⊗\tau_{K}^{-1}\right)}}^{\omega }}_{pq\cdots ,ij\cdots }={\left(\tau_{K}^{}\right)}_{p,i}{\left(\tau_{K}^{}\right)}_{q,j}\cdots$. In addition, we also implied summation over repeated indexes on the right-hand side of Equation (10). Note that the size of these matrices increases exponentially with cluster size ω, $K^{\omega }$x$K^{\omega }$; in particular, for ω=4, the matrices have 256 × 256 entries. The cluster probabilities are normalized as expressed in Equation (11)

(11) $$\sum_{\forall [(pq\cdots )]}^{K^{\omega }}y_{\omega ,n}^{\left(pq\cdots \right)}\left[\vec{\sigma }\right]=1.$$

As a consequence of the normalization of the cluster probabilities, it is possible to separate probabilities of the decorated sub-clusters from the probabilities of the maximal cluster by partial summations over all possible decorations for the sites that belong to the maximal cluster (ω,n) but not the i-th sub-cluster ($\omega_{i},n_{i}$):

(12) $$\sum_{\left(pq\cdots \right)\in \{\left[{\left(pq\cdots \right)}_{\omega_{i},n_{i}}\right]\}}^{}y_{\omega ,n}^{\left(pq\cdots \right)}\left[\vec{\sigma }\right]=y_{\omega_{i},n_{i}}^{\left(pq\cdots \right)}\left[\vec{\sigma }\right].$$

From Equation (10) and for the case with ω = 2, it follows that the generalized expression for SRO of species *p* and *q* at the nth shell, $\alpha_{2,n}^{\left(pq\right)}\left[\vec{\sigma }\right]\left(\;p\neq q\right)\;$, can be interpreted as the tendency to order or segregate species *p* and *q* with respect the disordered random probability given by the product of their elemental, *p*, bulk concentration $x_{p}\left[\vec{\sigma }\right]$. For the four component Cr-Fe-Mn-Ni system, there are six chemically distinct SRO parameters: $\alpha_{2,n}^{\left(01\right)}\left[\vec{\sigma }\right]$ for Cr-Fe; $\alpha_{2,n}^{\left(02\right)}\left[\vec{\sigma }\right]$ for Cr-Mn; $\alpha_{2,n}^{\left(03\right)}\left[\vec{\sigma }\right]$ for Cr-Ni; $\alpha_{2,n}^{\left(12\right)}\left[\vec{\sigma }\right]$ for Fe-Mn; $\alpha_{2,n}^{\left(13\right)}\left[\vec{\sigma }\right]$ for Fe-Ni; and $\alpha_{2,n}^{\left(23\right)}\left[\vec{\sigma }\right]$ for Mn-Ni. The SRO allows a quantitative description of the interactions between atoms as a function of temperature to predict order-disorder transition temperatures [11,29]. In particular, the matrix formalism from Equations (7) and (10) allows one to generalize the SRO treatment for an arbitrary number of components, *K*:

(13) $$y_{2,n}^{\left(pq\right)}\left[\vec{\sigma }\right]=x_{p}\left[\vec{\sigma }\right]x_{q}\left[\vec{\sigma }\right]\left(\;1-\alpha_{2,n}^{\left(pq\right)}\left[\vec{\sigma }\right]\right).\;$$

### 2.2. Configuration Entropy in the Matrix Formulation

In general, a thermodynamical system in state $\vec{\sigma }$ and with enthalpy of mixing given by the CE Hamiltonian $\Delta H_{CE}^{Mixing}\left[\vec{\sigma }\right]$ is described by a set of symmetry unique probability distributions $y_{\omega ,n}^{\left(pq\cdots \right)}\left[\vec{\sigma }\right]$ characterized by decorations of a chosen maximal (ω,n) cluster. In practice, the chosen maximal cluster (ω,n) contains few points. As a mean field approximation [27], the CVM can be rationalized as a factorization of the probability distributions of the (ω,n) maximal cluster into integer powers $\eta_{\omega_{1},n_{1}},\eta_{\omega_{2},n_{2}}\cdots ,\eta_{\omega_{s[\omega ,n]},n_{s[\omega ,n]}}$ of the probability distributions of the sub-clusters $\left(\omega_{i},n_{i}\right)\subseteq \left(\omega ,n\right);i\;=\;1,\cdots ,\left[\omega ,n\right]$ [19] with decorations ${\left(pq\cdots \right)}_{\omega_{i},n_{i}}$ corresponding to the components ${\left(pq\cdots \right)}_{\omega_{i},n_{i}}$ of all decorations (pq⋯)$\in \{{\left(pq\cdots \right)}_{\left(\omega ,n\right)}\}$ occupied by sites of the ($\omega_{i},n_{i}$) sub-cluster

(14) $$y_{\omega ,n}^{\left(pq\cdots \right)}\left[\vec{\sigma }\right]={\left(y_{\omega_{1},n_{1}}^{{\left(pq\cdots \right)}_{\omega_{1},n_{1}}}\left[\vec{\sigma }\right]\right)}^{\eta_{\omega_{1},n_{1}}}{\left(y_{\omega_{2},n_{2}}^{{\left(pq\cdots \right)}_{\omega_{2},n_{2}}}\left[\vec{\sigma }\right]\right)}^{\eta_{\omega_{2},n_{2}}}\cdots {\left(y_{\omega_{s[\omega ,n]},n_{s[\omega ,n]}}^{{\left(pq\cdots \right)}_{\omega_{s[\omega ,n]},n_{s[\omega ,n]}}}\left[\vec{\sigma }\right]\right)}^{\eta_{\omega_{s[\omega ,n]},n_{s[\omega ,n]}}}.$$

The following expression, a consequence of the CVM factorization scheme, can be derived from [19] and is the reason why the disordered configuration at high temperature is reproduced from multi-body probabilities, $\sum_{i=1}^{\left[\omega ,n\right]}\omega_{i}\eta_{\omega_{i},n_{i}}=-1$. This occurs because, at the high temperature limit, the CVM probabilities tend to products of composition of the species involved in the decorations of the cluster:

(15) $$y_{\omega_{i},n_{i}}^{{\left(pq\cdots \right)}_{\omega_{i},n_{i}}}\left[\vec{\sigma }\right]\underset{T\to \infty }{\to }\prod_{j=1}^{\omega_{i}}y_{1,1}^{{\left({\left(pq\cdots \right)}_{j}\right)}_{\omega_{i},n_{i}}}\left[\vec{\sigma }\right]=\prod_{j=1}^{\omega_{i}}x_{{\left({\left(pq\cdots \right)}_{j}\right)}_{\omega_{i},n_{i}}}\left[\vec{\sigma }\right],$$

where $y_{1,1}^{{\left({\left(pq\cdots \right)}_{j}\right)}_{\omega_{i},n_{i}}}\left[\vec{\sigma }\right]=x_{{\left(pq\cdots_{j}\right)}_{\omega_{i},n_{i}}}\left[\vec{\sigma }\right]$ is the concentration of the ${\left(pq\cdots \right)}_{j}$ equal to one of the integers 0,1,⋯,K−1 in the disordered state of the alloy. A natural consequence of the factorization scheme chosen for the CVM multi-body probabilities is that the configuration entropy assumes the formulation

(16) $$S_{\omega ,n}\left[\vec{\sigma }\right]\equiv \sum_{i=1}^{s[\omega ,n]}\eta_{\omega_{i},n_{i}}{\tilde{S}}_{\omega_{i},n_{i}}\left[\vec{\sigma }\right]=\sum_{i=1}^{s[\omega ,n]}\eta_{\omega_{i},n_{i}}\sum_{\forall [{\left(pq\cdots \right)}_{\omega_{i},n_{i}}]}^{K^{\omega_{i}}}k_{B}y_{\omega_{i},n_{i}}^{{\left(pq\cdots \right)}_{\omega_{i},n_{i}}}\left[\vec{\sigma }\right]ln\left(y_{\omega_{i},n_{i}}^{{\left(pq\cdots \right)}_{\omega_{i},n_{i}}}\left[\vec{\sigma }\right]\right),$$

where ${\tilde{S}}_{\omega_{i},n_{i}}$, is the entropy contribution to cluster (ω,n) from the sub-cluster $\left(\omega_{i},n_{i}\right)$:

(17) $${\tilde{S}}_{\omega_{i},n_{i}}\left[\vec{\sigma }\right]=\sum_{\forall {\left(pq\cdots \right)}_{\omega_{i},n_{i}}}^{K^{\omega_{i}}}k_{B}y_{\omega_{i},n_{i}}^{{\left(pq\cdots \right)}_{\omega_{i},n_{i}}}\left[\vec{\sigma }\right]ln\left(y_{\omega_{i},n_{i}}^{{\left(pq\cdots \right)}_{\omega_{i},n_{i}}}\left[\vec{\sigma }\right]\right).$$

The set of integers $\eta_{\omega_{1},n_{1}},\eta_{\omega_{2},n_{2}}\cdots ,\eta_{\omega_{s[\omega ,n]},n_{s[\omega ,n]}}$ are the mean field integer coefficients associated with the partition function of the alloy system. In the theory of regular mixtures, the coefficients can be found from the recursive heuristic expression after Kikuchi, [30], Barker [31] and whose formulation was explicitly derived by using group theoretic methods by Gratias et al. [32]. It requires the determination of two quantities: the site multiplicity $N_{\omega_{i},n_{i}}$ and the sub-cluster multiplicity $N_{\omega_{i},n_{i}}^{\beta }$. The site multiplicity can be determined by calculating the number of symmetry operators, $N_{\omega_{i},n_{i}}$, that stabilize the $\omega_{i},n_{i}$ cluster i.e., g∈G such that the application of *g* into the set of cluster positions $\left\{\vec{\tau_{1}},\vec{\tau_{2}},\cdots ,\vec{\tau_{\omega }}\right\}$ results in a permutation of the set. Then, $N_{\omega_{i},n_{i}}=\frac{\left|G\right|}{|N_{\omega_{i},n_{i}}|},$ where |G| is the order of the point group associated with the space group *G* and $|N_{\omega_{i},n_{i}}|$ is the order of the group $N_{\omega_{i},n_{i}}$. The sub-cluster multiplicity $N_{\omega_{i},n_{i}}^{\beta }$ is just the frequency of the cluster β that is contained in the cluster ($\omega_{i},n_{i}$)

(18) $$\eta_{\omega_{i},n_{i}}=-N_{\omega_{i},n_{i}}-\sum_{\left(\omega_{i},n_{i}\right)\subset \beta \subseteq \left(\omega ,n\right)}N_{\omega_{i},n_{i}}^{\beta }\eta_{\beta }.$$

In particular, the formulation applied to a point cluster retrieves the Bragg–Williams approximation for the maximal cluster (ω=1,n=1), i.e., a site cluster, giving the entropy weight of $\eta_{\omega_{1}=1_{1},n_{1}=1}=-1$ and for a 2-body cluster (ω=2,n) in the *n*th coordination shell, we get $\eta_{\omega_{2},n_{2}}=-N_{2,n}$ and $\eta_{\omega_{1},n_{1}}=2N_{2,n}-1$, where $N_{2,n}$ is site multiplicity of the cluster (2,n) calculated from the sites in this cluster and the space group *G*.

In this work, the above matrix formulation is applied in the hybrid CE-Monte Carlo method which performs the free energy minimization from the CE Hamiltonian in a combination with Monte Carlo simulations. Within the process, the Monte Carlo method produces the correlation functions for the equilibrium configurations found at each of the temperatures investigated. The hybrid approach uses these correlation functions in the analytic expressions for configuration entropy.

There is an alternative to the hybrid approach for the entropy calculation where the thermodynamic integration method can be used. Here, entropy is calculated from the configuration contribution to the specific heat at constant volume, $C_{conf}$ derived from the fluctuations of enthalpy of mixing at temperature values in a fine grid of temperature values:

(19) $$S_{conf}\left[T\right]=\int_{0}^{T}\frac{C_{conf}}{T^{\prime }}dT^{\prime }=\int_{0}^{T}\frac{\langle {\Delta H_{CE}^{Mixing}}^{2}\rangle -{\langle \Delta H_{CE}^{Mixing}\rangle }^{2}}{{T^{\prime }}^{3}}dT^{\prime }$$

where ${\langle \Delta H_{CE}^{Mixing}\rangle }^{2}$ and $\langle {\Delta H_{CE}^{Mixing}}^{2}\rangle$ are the square of the mean and mean square enthalpies of mixing, respectively, calculated by averaging over all the MC steps at the accumulation stage for a given temperature. The accuracy of evaluation of configuration entropy depends on the size of temperature integration step and the number of MC steps performed at the accumulation stage [33]. The integration of specific heat is performed from 0 K to the temperature *T*. In order to calculate the configuration entropy at a given temperature, the value for specific heats at lower temperatures is thus required. For example, assuming that the chosen temperature step is equal to 5 K, to evaluate configuration entropy numerically at 3000 K would require computing the specific heat at 600 smaller temperatures. In contrast, using Equation (17), the configuration entropy can be computed analytically from the correlation functions at any given temperature and alloy composition. Our experiences show that the computational time using the hybrid method can be of two orders faster than those by the thermodynamic integration.

### 2.3. Computational Details

The DFT enthalpies of mixing given by Equation (1) were calculated from fully relaxed spin-polarized DFT total energy calculations performed using the projector augmented wave (PAW) method [34] implemented in Vienna Ab initio Simulation Package (VASP) [35,36,37,38,39]. Exchange and correlation were treated in the generalized gradient approximation (GGA) and the Perdew-Burke-Ernzerhof (PBE) functional [40]. The core configurations of Fe, Cr, Mn and Ni in PAW potentials are [Ar]3d${}^{7}$4s${}^{1}$, [Ar]3d${}^{5}$4s${}^{1}$, [Ar]3d${}^{6}$4s${}^{1}$ and [Ar]3d${}^{9}$4s${}^{1}$, respectively. The total energies were calculated using the Monkhorst–Pack [41] mesh of 12 × 12 × 12 k-point for a four-atom FCC cubic cell. The plane wave cut-off energy used in the calculations was 400 eV. The total energy convergence criterion was set to 10${}^{-6}$ eV/cell, and force components were relaxed to 10${}^{-3}$ eV/nm.

In the semi canonical Monte Carlo calculations performed, the temperature range and temperature steps are important. The accuracy of the thermodynamic integration method to calculate configuration entropy generally requires a smaller temperature integration step, ΔT=5 K and also depends on the number of Monte Carlo passes [33]. In particular, we use a cell containing 2048 atoms distributed into a 8 × 8 × 8 primitive unit cell, and average compositions for the ensemble given by Cr${}_{18}$Fe${}_{27}$Mn${}_{27}$Ni${}_{28}$ and equiatomic Cr${}_{25}$Fe${}_{25}$Mn${}_{25}$Ni${}_{25}$. The Monte Carlo simulations were performed from random configurations at high temperature (3000 K) where the configuration entropy at the high-temperature limit is given by $k_{B}ln\left(4\right)$ for equiatomic and $-k_{B}\left\{0.18*ln\left(0.18\right)+2*0.27*ln\left(0.27\right)+0.28*ln\left(0.28\right)\right\}$ for Cr${}_{18}$Fe${}_{27}$Mn${}_{27}$Ni${}_{28}$ [42]. By quenching down systematically with the temperature step of Δ T = 5 K, various equilibrium configurations were obtained at lower temperatures. For thermodynamic integration calculation of configuration entropy, we integrated numerically the specific heat at constant volume using the theoretical formula (19) starting from the lowest temperature value 0 K to 3000 K.

## 3. Cluster Probability Functions in FCC Cr-Fe-Mn-Ni Alloys

We apply the matrix formulation of cluster expansion outlined in Section 2.1 to the FCC CrFeMnNi system for investigating the temperature and composition dependent cluster probability distribution functions and the configuration entropy.

### 3.1. Cluster Expansion Hamiltonian for FCC CrFeMnNi

The DFT enthalpies of mixing for the FCC CrFeMnNi system were used to map iteratively into the cluster expansion Hamiltonian given by Equation (2) using the ATAT package [16]. The mapping has been performed systematically from the six binary and four ternary constituent subsystems of the considered quaternary. The database of structures for the cluster expansion consisted of 835 structures categorized by the difference of local environments in binaries (structures with two chemical elements: 58 CrFe, 55 CrMn, 77 CrNi, 58 FeMn, 54 FeNi and 52 MnNi), ternaries (structures with three chemical elements: 89 CrFeMn, 85 CrFeNi, 46 FeMnNi, and 66 CrMnNi); and 191 quaternaries CrFeMnNi. More information about the type of binary and ternary structures used in our DFT database has been detailed in our previous work [17]. Structures are typically ordered ones with their composition ranging for each constituent element from 5% to 95%. It is important to stress here that, different to other studies of HEAs, our DFT database for constructing the CE Hamiltonian didn’t include randomly distributed structures such as the so-called special quasi-random structures (SQSs). The latter, however, can be generated within the present approach from Monte-Carlo simulations after obtaining the reliable ECIs. The set of clusters which have minimized the cross-validation score of 12.95 meV/atom, consists of six 2-body, two 3-body and one 4-body ECIs. In the present work, the clusters with the same sizes and relative positions have been included consistently in the considered subsystems. The prediction of the corresponding ground-state intermetallic phases is in a good agreement with the experimental binary phase diagrams available (for example, the ferromagnetic $FeNi_{3}$ in $L1_{2}$ structure and anti-ferromagnetic MnNi in $L1_{0}$ structure) as well as with the previous theoretical study for the ternary and ferrimagnetic $CrFe_{2}Ni$ phase in $NiCu_{2}Zn$ structure [17]. A new intermetallic phase $FeCr_{2}MnNi_{4}$ is predicted for the quaternary system and full results of magnetic properties in CrFeMnNi systems will be discussed in a separate work.

The CE Hamiltonian for the quaternary system CrFeMnNi consists, in total, of 83 different decorated clusters distributed among 10 different non-decorated clusters: four decorated clusters for the point cluster, (ω=1,n=1); six decorated clusters for each pair cluster (ω=2,n=1,⋯,6); 10 decorated clusters for the non-decorated cluster (ω=3,n=1); 18 decorated clusters for the non-decorated cluster (ω=3,n=2); and 15 decorated clusters for the non-decorated cluster (ω=4,n=1). The decoration labels, (s) required to specify the clusters, are listed in Table 1. Each non-decorated cluster is defined by the coordinates (specified with respect to the standard Cartesian coordinate system in units of lattice spacing) of the lattice sites that it includes. The decorations define the chemical species allocated to each site in strictly the same order i.e., for (ω=2,n=1) cluster the decoration (2,3) means that species 2 is allocated for site with coordinates (1,1,1) and species 3 is allocated for site with coordinates (1/2,3/2,3/2).

### 3.2. Full Set of Cluster Decorations

In general, the set of temperature dependent decorated cluster correlation functions, $\langle \Gamma_{\omega ,n}^{\left(s\right)}\left[\vec{\sigma }\right]\rangle$, constitute a set of $K^{\omega }$ quantities for a given maximal cluster (ω,n) in the CE. From each of the $K^{\omega }$ temperature dependent decorated cluster correlation functions, the matrix formalism described in Section 2.1 generates the set of temperature dependent multi-body probability functions describing the temperature dependent behavior associated with the maximal cluster (ω,n). The symmetry unique decorations for all of the clusters in the CE, ∀(ω,n), are reported in the ATAT *clusters* output file and here they are listed in Table 1. If one cluster is included into another, it becomes a sub-cluster $\left(\omega_{i},n_{i}\right)\in \left(\omega ,n\right)$; these sub-clusters have been classified according to their inclusion into maximal clusters, and are listed in Table 2. If the sub-cluster $\left(\omega_{i},n_{i}\right)$ is included in the maximal cluster, $\left(\omega_{i},n_{i}\right)\in \left(\omega ,n\right)$, then the decorations from the sub-cluster ${\left(s\right)}_{\left(\omega_{i},n_{i}\right)}$ can be transferred into the decorations of the maximal cluster, ${\left(s\right)}_{\left(\omega ,n\right)}$, by using the intrinsic space group symmetry of the parent lattice. In general, any given sub-cluster $\left(\omega_{i},n_{i}\right)$ can be found several times within the cluster (ω,n). For convenience, symmetry of the cluster decorations is implemented by means of permutation operators in Table 2 including X for the empty site. The full set of decorated cluster correlation functions, $\langle \Gamma_{\omega ,n}^{\left(s\right)}\left[\vec{\sigma }\right]\rangle$, corresponding to the maximal cluster (ω,n), is generated by studying which of the decorations ${\left(s\right)}_{\omega_{i},n_{i}}$ form the sub-clusters $\left(\omega_{i},n_{i}\right)$ in (ω,n). The decorations can have empty cluster, i.e., at least one integer entry in $0\subseteq {\left(s\right)}_{\omega_{i},n_{i}}$.

For the case of the maximal cluster (ω=2,n=1) consisting of sites {(1,1,1),(1,3/2,3/2)}, see Table 1, we have a full set of $4^{2}=16$ 2-tuples of decorations, each associated with a temperature dependent decorated cluster correlation function. It can be noted that the cluster {(1,1,1),(1,3/2,3/2)} contains two sub-clusters: the point cluster {(1,1,1)} that has decorations {(0),(1),(2),(3)}; and the cluster itself which has decorations {(1,1),(2,1),(3,1),(2,2),(2,3),(3,3)}. For each of these decorations, there are corresponding decorated cluster correlation functions. In this case, the point cluster is contained twice: once in (1,1,1)⊂{(1,1,1),(1,3/2,3/2)} and also in (1,3/2,3/2)⊂{(1,1,1),(1,3/2,3/2)}. Similarly the cluster itself {(1,1,1),(1,3/2,3/2)} is contained once. Thus a point cluster decorated by (s)=(3) is transfered to ω=2-tuples notation in the maximal cluster as (3,0) or (0,3). This can be captured by the permutation operators {(1,X),(X,1)} (see Table 2) where *X* stands for an empty cluster where a 0 should be placed. Similarly, the decorations denoted by (s)=(1,2) in the 2-tuples notation are equivalent to the symmetry equivalent decorations (s)=(1,2) and $\left(s^{\prime }\right)=\left(2,1\right)\equiv \left(1,2\right)$. The symmetry effect can again be captured by permutation operators {(1,2),(2,1)}, where *X* no longer appears, since the $\omega_{i}=2$-tuples have the same order ($\omega =\omega_{i}$) as the ω=2-tuples from the maximal cluster (ω=2,n=1). The analysis outlined above for the 2-body cluster {(1,1,1),(1,3/2,3/2)} with K=4 components can be extended to the remaining (ω=2,n>1) maximal clusters. The permutation operators that carry out the appropriate decorations ${\left(s\right)}_{\omega_{i},n_{i}}$ of sub-clusters $\left(\omega_{i},n_{i}\right)$ into (ω,n) cluster ω-tuples notation are detailed in Table 2. It should be noted that, for any 2-body cluster, the point and corresponding pair sub-clusters add up, forming 4+6=10 symmetrically unique correlation functions $\langle \Gamma_{\omega ,n}^{\left(ij\right)}\left[\vec{\sigma }\right]\rangle$.

Referring to the maximal cluster (ω=3,n=1), from Table 2, there are three contained sub-clusters $\left(\omega_{1}=1,n_{1}=1\right)$, $\left(\omega_{2}=2,n_{2}=1\right)$ and $\left(\omega_{3}=3,n_{3}=1\right)$ within the maximal cluster (ω=3,n=1). The point sub-cluster $\left(\omega_{1}=1,n_{1}=1\right)$ has, according to Table 1, four decorations associated with it; the 2-body cluster $\left(\omega_{2}=2,n_{2}=1\right)$ has six decorations associated with itself; and finally the sub-cluster $\left(\omega_{3}=3,n_{3}=1\right)$ has 10 decorations associated with itself. The total number of symmetry unique decorated cluster correlation functions, which is smaller than $K^{\omega }=4^{3}=64$, but can nevertheless fully describe cluster (ω=3,n=1) with K=4 components, is therefore given by 4+6+10=20 decorated cluster correlation functions. From these 20 symmetry unique decorated cluster correlations, it is possible to generate a total of 64 decorated cluster correlation functions by using the permutation operators in Table 2. The number of permutation operators to transfer decorations from sub-cluster $\left(\omega_{1}=1,n_{1}=1\right)$ to (ω=3,n=1), is 3; from $\left(\omega_{2}=2,n_{2}=1\right)$, six permutation operators, and, from the cluster itself $(\omega_{3}=3,n_{3}=1),$ there are three permutation operators. The total number of permutation operators is therefore 3+6+6=15. The remaining decorations, which are not listed in Table 1, add up to 64−20=44 and are obtained by using appropriately the 15 permutation operators corresponding to the maximal cluster (ω=3,n=1). Similarly, the maximal cluster (ω=3,n=2) has 34 symmetrically unique decorations and 12 permutation operators; and (ω=4,n=1) has 35 symmetry unique decorations with 64 permutation operators (all possible permutations for K=4).

### 3.3. Four-Body Probability Functions from Monte Carlo Simulations

After performing semi-canonical Monte Carlo simulations for alloy compositions Cr${}_{18}$Fe${}_{27}$Mn${}_{27}$Ni${}_{28}$ and equiatomic Cr${}_{25}$Fe${}_{25}$Mn${}_{25}$Ni${}_{25}$, we use the correlation functions calculated at each of the temperatures to study the temperature dependent probabilities of decorated clusters corresponding to the equilibrium configuration $\left[\vec{\sigma }\right]$ of the Monte Carlo super-cell following the formalism described in the Methods section. For each non-decorated cluster ω,n and temperature, we generate the corresponding $\tau^{-1}$ matrices with dimension $K^{\omega }$x$K^{\omega }$ and a vector formed by all the decorated correlation cluster functions, $\langle \Gamma_{\omega ,n}^{\left(ij\cdots \right)}\left[\vec{\sigma }\right]\rangle$ with dimension $K^{\omega }$. The Monte Carlo simulations output only the symmetrically unique correlation functions (see Table 1), which are less than the $K^{\omega }$ required for matrix operation. In order to generate the full set of $K^{\omega }$ from the symmetrically unique correlation functions, we devise a set of permutation operators $g_{i}$ (see Table 2) that indicate how an arbitrary decoration ω-tuple of integers is obtained from the symmetrically unique decorations belonging to the non-decorated cluster or one of its sub-clusters (see the third column of Table 2 for identifying the cluster-sub-cluster relation). Table 2 contains all the operators necessary to generate cluster probabilities, $y_{\omega ,n}^{\left(s\right)}$, for any possible decoration, (s), of 10 non-decorated clusters employed in the cluster expansion Hamiltonian.

**Figure 1:**
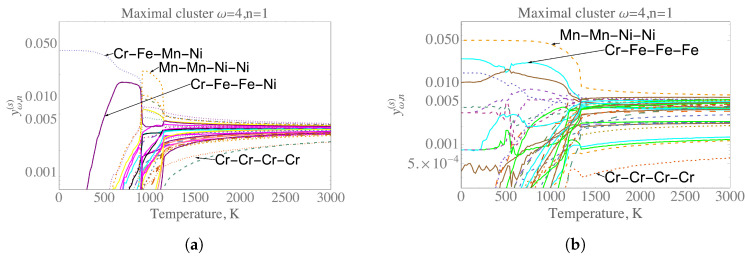
4-body probabilities obtained from the hybrid Cluster Expansion (CE)-Monte Carlo calculations. (**a**) all the 4-body probabilities for the equiatomic composition Cr${}_{25}$Fe${}_{25}$Mn${}_{25}$Ni${}_{25}$ as a function of temperature; (**b**) the same as in (**a**) but for the composition Cr${}_{18}$Fe${}_{27}$Mn${}_{27}$Ni${}_{28}$.

As an important case of study, we chose the maximal cluster given by (ω = 4, *n* = 1) to obtain the 4-body configuration probabilities. Figure 1a,b show the plots of 35 symmetrically unique probabilities for the 4-body maximal cluster defined by the set of lattice sites ((1,1,1),(3/2,3/2,1),(3/2,1,1/2),(1,3/2,1/2)) as a function of temperature.

The most important 4-body bonding configurations in the temperature range from 0 to 3000 K for the two alloy systems (the equiatomic Cr${}_{25}$Fe${}_{25}$Mn${}_{25}$Ni${}_{25}$ and Cr${}_{18}$Fe${}_{27}$Mn${}_{27}$Ni${}_{28}$) are highlighted in Figure 1a,b. It should be noted that, for both compositions, the cluster configuration referred to as Cr-Cr-Cr-Cr appears as the least probable of all the cluster configurations. This finding is consistent with the fact that Cr has the BCC ground-state and therefore the probability of finding a Cr cluster in the FCC lattice is negligible, in particular in the low temperature region.

For the equiatomic composition, the probability of Cr-Fe-Mn-Ni is particularly very high at temperatures between 0–900 K. The presence of 4-body Cr-Fe-Mn-Ni clusters at low temperatures demonstrates the relationship with our DFT/CE prediction of the new ordered phase $FeCr_{2}MnNi_{4}$ in the quaternary system. Furthermore, in the temperature range below 900 K, the cluster configuration Cr-Fe-Fe-Ni shows that it is the second most probable configuration. This configuration is directly correlated with the ordered $Fe_{2}CrNi$ structure predicted in our earlier study of phase stability in the ternary Fe-Cr-Ni system [17]. In the temperature region between 900 K and 1200 K, an increase of probability of Mn-Mn-Ni-Ni cluster is significantly important. Beyond 1200 K, all of the cluster configuration probabilities tend to the solid solution or random configuration.

For the system with average composition Cr${}_{18}$Fe${}_{27}$Mn${}_{27}$Ni${}_{28}$, the cluster probability of decorations given by Mn-Mn-Ni-Ni appears to dominate until 1300 K, where the solid solution or random configuration begins. Furthermore, the second most probable cluster configuration in the temperature range 500–1200 K appears to be Cr-Fe-Fe-Fe. Again, these findings are remarkable and the origin of these clusters would need a more detailed discussion.

**Figure 2:**
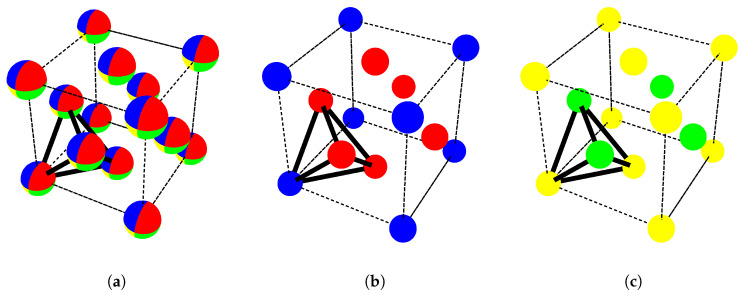
(**a**) most probable phase at high temperature (disordered structure); (**b**,**c**): two most probable ordered phases at low temperature in the equiatomic Cr${}_{25}$Fe${}_{25}$Mn${}_{25}$Ni${}_{25}$ and Cr${}_{18}$Fe${}_{27}$Mn${}_{27}$Ni${}_{28}$ HEAs compositions. Cr, Fe, Mn and Ni are illustrated in blue, red, yellow and green respectively. (**a**) A1 phase, sites are occupied by Cr, Mn, Fe, and Ni in probabilities determined by their average concentration in the system; (**b**) L1${}_{2}$ phase corresponding to CrFe${}_{3}$ with Cr and Fe; (**c**) L1${}_{0}$ phase corresponding to MnNi with Mn and Ni.

The prediction of the high probabilities for Mn-Mn-Ni-Ni and Cr-Fe-Fe-Fe clusters in Cr${}_{18}$Fe${}_{27}$Mn${}_{27}$Ni${}_{28}$ alloy composition shown in Figure 1b can be explained by the decoration of the first nearest neighbor four-atom cluster interaction obtained from the CE Hamiltonian. It is important to stress that, in this case, the CE method reproduces the well-known result from the tetrahedron approximation in the CVM [18,19]. The configuration Mn-Mn-Ni-Ni is understood to be related to L1${}_{0}$ structure in Strukturbericht notation with Mn and Ni atoms, whereas the composition Cr-Fe-Fe-Fe is related the L1${}_{2}$ structure in Strukturbericht notation with Cr and Fe atoms. Both of these structures are depicted in Figure 2 and they are in a full agreement with our first-principles investigations. Indeed, the MnNi-$L1_{0}$ structure is predicted by both the DFT and CE Hamiltonian to be one of the ground-state structures not only for the binary Mn-Ni system but also for the quaternary Fe-Cr-Mn-Ni one. The $CrFe_{3}$-$L1_{2}$ structure has also been found as the lowest-energy binary structure in the previous study of the phase stability of Fe-Cr binary in the FCC lattice (see Figure 2a from [17]). In particular, the latter structure has also been found to be stable due to the strong anti-ferromagnetic interaction between Cr and Fe from the magnetic cluster expansion (MCE) performed for the ternary CrFeNi system [43]. In that work, Monte Carlo simulations using MCE indicated that ordered magnetic structures in the Ni-rich corner (ferromagnetic) of the FeCrNi system persists until temperatures of 600 K, and that for the atomic compositions between CrFe${}_{2}$ and CrFe${}_{3}$ the anti-ferromagnetic order was retained beyond 500 K.

## 4. Configuration Entropy in a Cr-Fe-Mn-Ni System

For each temperature value in the range 0–3000 K, the configuration probabilities corresponding to cluster (ω=4,n=1) are used in Equation (17) to obtain the quantities ${\tilde{S}}_{\omega_{i},n_{i}}\left[\vec{\sigma }\right]$. There is one quantity ${\tilde{S}}_{\omega_{i},n_{i}}\left[\vec{\sigma }\right]$ and a corresponding weight $\eta_{\omega_{i},n_{i}}$ for each of the ($\omega_{i},n_{i}$) sub-clusters: (ω=1,n=1), (ω=2,n=1); (ω=3,n=1); and (ω=4,n=1) included in the maximal cluster (ω=4,n=1). The total configuration entropy, $S_{\omega ,n}\left[\vec{\sigma }\right]$, is calculated by adding the $\eta_{\omega_{i},n_{i}}$ weighted quantities ${\tilde{S}}_{\omega_{i},n_{i}}\left[\vec{\sigma }\right]$. By applying the same arguments developed for the cluster (ω=4,n=1), the total configuration entropy for each of the 10 different maximal clusters can be calculated. The sub-clusters and the permutation operators contained in each of the 10 maximal clusters appearing in the cluster expansion are detailed in Table 2.

**Figure 3:**
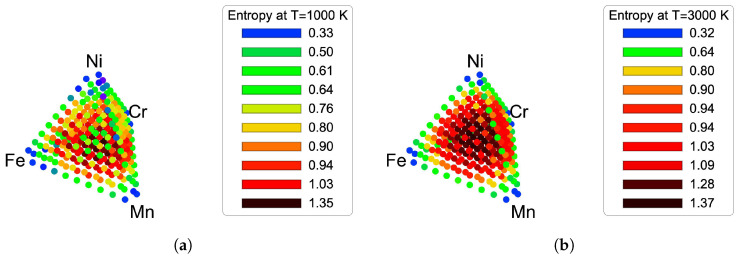
Composition dependent entropies obtained from Monte Carlo simulations in CE. (**a**) Composition dependent entropy at fixed temperature 1000 K; (**b**) Composition dependent entropy at fixed temperature 3000 K.

**Figure 4:**
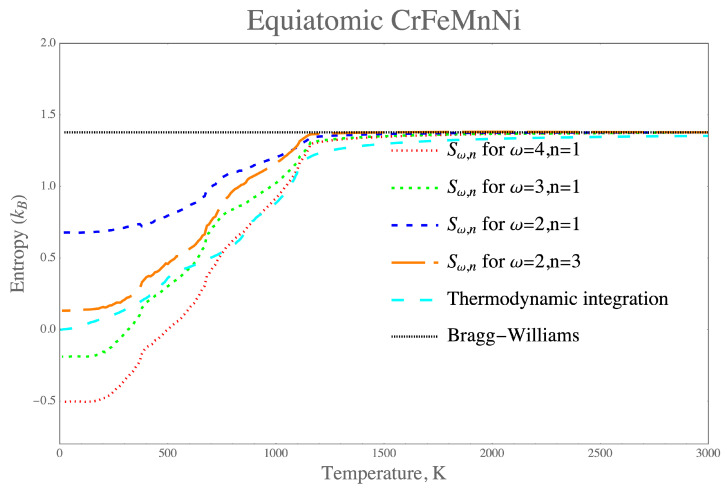
Temperature dependence of configuration entropy evaluated at various levels of cluster approxinations and compared with the thermodynamic integration result at the equiatomic composition Cr${}_{25}$Fe${}_{25}$Mn${}_{25}$Ni${}_{25}$.

Composition dependent entropies at fixed temperatures 1000 K and 3000 K are shown in Figure 3a,b. At each of these temperature values, any of the configuration entropy expressions from Table 2 provides approximately the same value. At 3000 K, the entropy is maximized in the center of the tetrahedron, namely at the equiatomic composition to 1.37 $k_{B}$, and it is decreased upon lowering the temperature at all of the composition points. The variation of configuration entropy is too complex to be captured in the fixed temperature plots, therefore we use specifically the equiatomic composition and calculate the configuration entropy as a function of temperature in Figure 4. Regarding the high temperature limit value of entropy in Figure 4, we use as maximal cluster that from the 4-body, 3-body and 2-body in the nearest neighbor cluster probabilities. Above 1200 K, the cluster approximation for the configuration entropy approaches the high temperature limit of disordered solute solution ($-k_{B}$$\sum_{p}^{4}x_{p}\left[\vec{\sigma }\right]ln\left(x_{p}\left[\vec{\sigma }\right]\right)$) much more rapidly than the thermodynamic integration. The reason the high temperature limit is preserved can be clearly seen from the factorization of the CE expression that resulted from analytical derivation in Equation (15). It is important to stress that, within the high-temperature limit, our theoretical prediction recovers the ideal configuration entropy of mixing for solution phase as it has been originally proposed by the phenomenological guidelines in designing high-entropy alloys using thermodynamic and topological parameters of the constituent elements [44].

It is worth mentioning that the entropy increase (Figure 4) as a function of temperature for the equiatomic alloy composition can thus be understood by the corresponding decrease in probabilities (Figure 1) of the ordered state of the structures L1${}_{0}$ towards the disorder configuration A1 in Figure 2. Similarly, in the alloy with composition Cr${}_{18}$Fe${}_{27}$Mn${}_{27}$Ni${}_{28}$, the increase of configuration entropy is correlated with the decrease in high probability of the configurations of Cr-Fe-Fe-Fe and Mn-Mn-Ni-Ni clusters originally related to the ordered FCC-like L1${}_{2}$-CrFe${}_{3}$ and L1${}_{0}$-MnNi structure, respectively.

For the cluster (ω=4,n=1), the calculated configuration entropy in Figure 4 appears to be negative at low temperature with respect to the positive values correctly predicted by the thermodynamic integration method. This demonstrates clearly that this maximum cluster within the tetrahedron approximation conventionally adopted within the CVM is only valid to describe the configuration entropy for the four-component Cr-Fe-Mn-Ni system in the high-temperature limit. It is shown from Figure 4 that the first nearest-neighbor triangle cluster (ω=3,n=1), which plays an important role within the tetrahedron approximation, also gives physically incorrect and negative entropy contribution at low temperature. As it has been discussed in the introduction, in difference from the CVM tetrahedron approximation, besides the first nearest-neighbor cluster contributions, the present CE results also include other pair clusters up to the sixth nearest neighbors and the second nearest-neighbor triangle cluster contributions. For example, in Figure 4, the third nearest-neighbor pair cluster (ω=2,n=3) gives the significantly positive contribution to configuration entropy in the entire range of temperature. These additional cluster contributions, in turn, ensure the correct behavior of the configuration entropy obtained from the thermodynamic integration. Therefore, from a statistical physics point of view, the hybrid technique combining Monte Carlo simulations with the CE method can be considered to be more advanced than the mean-field approach advocated within the CVM.

## 5. Conclusions

In this work, we develop a matrix formalism to study multi-body ordering probabilities beyond pair approximation previously used for investigating the SRO and configuration entropy in multi-component alloys by using a hybrid combination of CE and Monte Carlo methods. The cluster probabilities are worked out by explicit inversion within the orthonormal sets of the point functions adopted in the ATAT package and a direct product of a matrix formulation obtained from symmetrically independent correlation functions. The correlation functions are determined from semi-canonical Monte Carlo simulations and ECIs derived from DFT calculations. We apply our method to the quaternary FCC Cr-Fe-Mn-Ni system by considering 285 different alloy compositions covering the compositional space of the quaternary alloy as a function of temperature. To further assess our formulated expressions for configuration entropy, focus is put on two alloy compositions, equiatomic Cr${}_{25}$Fe${}_{25}$Mn${}_{25}$Ni${}_{25}$ and Cr${}_{18}$Fe${}_{27}$Mn${}_{27}$Ni${}_{28}$, to obtain cluster probabilities and understand the variation in configuration entropy with temperature due to the lowering of the ordering probability corresponding to the ordered configuration. The cluster probability plots against temperature show that, for the composition Cr${}_{18}$Fe${}_{27}$Mn${}_{27}$Ni${}_{28}$, there is a high probability of formation of the L1${}_{0}$ MnNi phase at low temperatures below 1300 K and for the L1${}_{2}$ CrFe${}_{3}$ phase in the temperature range 500–1200 K. Similarly, for the equiatomic composition, the L1${}_{0}$ MnNi phase appears stable in the temperature range 900–1200 K, while the lower temperature region is preferred for the configuration Cr-Fe-Mn-Ni. The configuration Cr-Cr-Cr-Cr was found to be the least probable configuration at all temperature ranges for both compositions of the Cr-Fe-Mn-Ni system. Furthermore, the configuration entropy as a function of temperature was derived from these probabilities: the high-temperature limit is in accordance with random solid solution approximation, but, at low temperatures, the entropy is seen to be reduced due to ordering or segregation tendencies which are in turn determined by multi-body probability functions including chemical short-range order.

We believe that the present study will help to promote further understanding of derivative phases as a function of temperature and multi-component alloy composition from disordered solid solutions in the phenomenological description of HEAs. By applying the formalism to the Cr-Fe-Mn-Ni system, it will also serve as a benchmarking example in designing radiation tolerant materials for advanced nuclear reactor systems by using the ab initio based CE method. The composition Cr${}_{18}$Fe${}_{27}$Mn${}_{27}$Ni${}_{28}$ multicomponent system has been studied for advanced nuclear applications due to promising irradiation resistance with regards to void swelling.

## Figures and Tables

**Table 1:** ω, highest coordination shell *n*, decoration (s) and coordinates of points in the relevant clusters on the Face-Centered Cubic (FCC) lattice. The coordinates are referred to the simple cubic Bravais lattice. Index (s) is the same as the sequence of points in the relevant cluster. The canonical order for decoration indexes, ${\left(s\right)}_{i}$, is 0, 1, 2 and 3 is Cr, Fe, Mn and Ni. All values of the Effective Cluster Interactions (ECIs) obtained from the present CE study are shown in the last column.

ω	*n*	(s)	Coordinates	ECI (meV/atom)	ω	*n*	(s)	Coordinates	ECI (meV/atom)
1	1	(0)	(1,1,1)	+0.00	3	1	(1,1,1)	(1,1,1)	+0.20
		(1)		+0.11			(2,1,1)	(3/2,1,1/2)	+0.90
		(2)		−0.04			(3,1,1)	(1,3/2,1/2)	+1.60
		(3)		−0.01			(2,2,1)		−3.40
2	1	(1,1)	(1,1,1)	+9.40			(3,2,1)		−0.50
		(2,1)	(1,3/2,3/2)	−0.10			(3,3,1)		+1.20
		(3,1)		+3.40			(2,2,2)		+0.20
		(2,2)		+0.40			(3,2,2)		+2.00
		(3,2)		+1.30			(3,3,2)		−0.50
		(3,3)		+6.00			(3,3,3)		+0.00
2	2	(1,1)	(1,1,1)	−9.20	3	2	(1,1,1)	(1,1,1)	−0.60
		(2,1)	(1,1,0)	+0.40			(2,1,1)	(1,3/2,1/2)	+1.00
		(3,1)		−4.40			(3,1,1)	(1,1,0)	+0.90
		(2,2)		−11.60			(1,2,1)		−1.80
		(3,2)		−3.50			(2,2,1)		+2.30
		(3,3)		−8.80			(3,2,1)		−0.70
2	3	(1,1)	(1,1,1)	0.90			(1,3,1)		−2.10
		(2,1)	(2,3/2,3/2)	2.60			(2,3,1)		−0.30
		(3,1)		3.50			(3,3,1)		−0.60
		(2,2)		1.60			(2,1,2)		−6.30
		(3,2)		−0.30			(3,1,2)		−1.20
		(3,3)		0.10			(2,2,2)		-0.60
2	4	(1,1)	(1,1,1)	−0.40			(3,2,2)		−0.10
		(2,1)	(2,1,2)	2.40			(2,3,2)		+1.60
		(3,1)		1.20			(3,3,2)		+0.60
		(2,2)		0.50			(3,1,3)		−2.00
		(3,2)		0.60			(3,2,3)		+0.30
		(3,3)		−0.80			(3,3,3)		−1.40
2	5	(1,1)	(1,1,1)	−1.00	4	1	(1,1,1,1)	(1,1,1)	−3.30
		(2,1)	(1,3/2,−1/2)	−3.50			(2,1,1,1)	(3/2,3/2,1)	+2.00
		(3,1)		−2.00			(3,1,1,1)	(3/2,1,1/2)	+0.70
		(2,2)		0.20			(2,2,1,1)	(1,3/2,1/2)	−2.90
		(3,2)		0.90			(3,2,1,1)		+0.60
		(3,3)		0.40			(3,3,1,1)		+0.70
2	6	(1,1)	(1,1,1)	0.80			(2,2,2,1)		−0.60
		(2,1)	(2,2,0)	2.10			(3,2,2,1)		−1.10
		(3,1)		1.00			(3,3,2,1)		+1.30
		(2,2)		−2.70			(3,3,3,1)		+2.60
		(3,2)		−1.30			(2,2,2,2)		−0.50
		(3,3)		0.50			(3,2,2,2)		+4.90
							(3,3,2,2)		+1.00
							(3,3,3,2)		−1.60
							(3,3,3,3)		−1.70

**Table 2:** List of permutation operators for generating the full set of decorations represented by $K^{\omega }$ dimensional integer arrays with entries taking values from 0 to K−1. The symmetry operators (see Equation (5)) represented here in permutation form in column 2 act on the set of 83 symmetry unique decorations indicated under the (s) column in Table 1 by permuting the entries in (s) or by introducing the empty cluster *X* for sub-clusters belong to a given cluster; examples with discussion are provided in Section 3.2. The space group of the disordered FCC structure, $g\in O_{h}^{5}$, is implicitly assumed in order to convolute the symmetry unique into the full set of decorations. The last four columns represent $\left(\omega_{i},n_{i}\right)$ *i*th sub-cluster of the maximal cluster (ω,n); $N_{\omega_{i},n_{i}}$ site multiplicity of ($\omega_{i},n_{i}$); $N_{\omega ,n}^{\omega_{i},n_{i}}$ sub-cluster multiplicity of the cluster $\left(\omega_{i},n_{i}\right)\in \left(\omega ,n\right)$; and $\eta_{\omega_{i},n_{i}}$, the sub-cluster ($\omega_{i},n_{i}$) contribution to configuration entropy expression corresponding to the maximal cluster (ω,n)

Maximal Cluster	Permutation Operators $\left\{g_{1},g_{2},\cdots \right\}$	Sub-Cluster $\left\{\omega_{i},n_{i}\right\}$	$N_{\omega_{i},n_{i}}$	$N_{\omega ,n}^{\omega_{i},n_{i}}$	$\eta_{\omega_{i},n_{i}}$
ω=1,n=1	{(1)}	$\omega_{1}=1,n_{1}=1$	1	1	−1
ω=2,n=1	{(1,X),(X,1)}	$\omega_{1}=1,n_{1}=1$	1	2	11
	{(1,2),(2,1)}	$\omega_{2}=2,n_{2}=1$	6	1	−6
ω=2,n=2	{(1,X),(X,1)}	$\omega_{1}=1,n_{1}=1$	1	2	5
	{(1,2),(2,1)}	$\omega_{2}=2,n_{2}=2$	3	1	−3
ω=2,n=3	{(1,X),(X,1)}	$\omega_{1}=1,n_{1}=1$	1	2	23
	{(1,2),(2,1)}	$\omega_{2}=2,n_{2}=3$	12	1	−12
ω=2,n=4	{(1,X),(X,1)}	$\omega_{1}=1,n_{1}=1$	1	2	11
	{(1,2),(2,1)}	$\omega_{2}=2,n_{2}=4$	6	1	−6
ω=2,n=5	{(1,X),(X,1)}	$\omega_{1}=1,n_{1}=1$	1	2	23
	{(1,2),(2,1)}	$\omega_{2}=2,n_{2}=5$	12	1	−12
ω=2,n=6	{(1,X),(X,1)}	$\omega_{1}=1,n_{1}=1$	1	2	7
	{(1,2),(2,1)}	$\omega_{2}=2,n_{2}=6$	4	1	−4
ω=3,n=1	{(1,X,X),(X,X,1),(X,1,X)}	$\omega_{1}=1,n_{1}=1$	1	3	−13
	{(1,2,X),(2,1,X),(2,X,1),(1,X,2),(X,1,2),(X,2,1)}	$\omega_{2}=2,n_{2}=1$	6	3	18
	{(1,3,2),(3,2,1),(2,1,3),(3,1,2),(2,3,1),(1,2,3)}	$\omega_{3}=3,n_{3}=1$	8	1	−8
ω=3,n=2	{(1,X,X),(X,X,1),(X,1,X)}	$\omega_{1}=1,n_{1}=1$	1	3	−19
	{(2,1,X),(1,2,X)}	$\omega_{2}=2,n_{2}=2$	1	1	9
	{(1,X,2),(2,X,1),(X,1,2),(X,2,1)}	$\omega_{3}=2,n_{3}=1$	6	2	18
	{(3,1,2),(1,3,2),(1,2,3)}	$\omega_{4}=3,n_{4}=2$	12	1	−12
ω=4,n=1	{(1,X,X,X),(X,X,X,1),(X,X,1,X),(X,1,X,X)}	$\omega_{1}=1,n_{1}=1$	1	4	−5
	{(X,X,1,2),(X,X,2,1),(1,2,X,X),(2,1,X,X)	$\omega_{2}=2,n_{2}=1$	6	6	6
	(X,1,X,2),(X,2,X,1),(2,X,1,X),(1,X,2,X)	$\omega_{2}=2,n_{2}=1$			
	(1,X,X,2),(2,X,X,1),(X,1,2,X),(X,2,1,X)}	$\omega_{2}=2,n_{2}=1$			
	{(1,3,2,X),(3,1,X,2),(2,X,1,3),(X,2,3,1)	$\omega_{3}=3,n_{3}=1$	8	4	0
	(3,2,1,X),(1,X,3,2),(X,1,2,3),(2,3,X,1)	$\omega_{3}=3,n_{3}=1$			
	(2,1,3,X),(X,3,1,2),(1,2,X,3),(3,X,2,1)	$\omega_{3}=3,n_{3}=1$			
	(2,X,3,1),(X,2,1,3),(1,3,X,2),(3,1,2,X)	$\omega_{3}=3,n_{3}=1$			
	(1,X,2,3),(3,2,X,1),(2,3,1,X),(X,1,3,2)	$\omega_{3}=3,n_{3}=1$			
	(3,X,1,2),(1,2,3,X),(X,3,2,1),(2,1,X,3)}	$\omega_{3}=3,n_{3}=1$			
	{(1,4,3,2),(4,1,2,3),(3,2,1,4),(2,3,4,1)	$\omega_{4}=4,n_{4}=1$	2	1	−2
	(4,3,1,2),(1,2,4,3),(2,1,3,4),(3,4,2,1)	$\omega_{4}=4,n_{4}=1$			
	(3,1,4,2),(2,4,1,3),(1,3,2,4),(4,2,3,1)	$\omega_{4}=4,n_{4}=1$			
	(3,2,4,1),(2,3,1,4),(1,4,2,3),(4,1,3,2)	$\omega_{4}=4,n_{4}=1$			
	(1,2,3,4),(4,3,2,1),(3,4,1,2),(2,1,4,3)	$\omega_{4}=4,n_{4}=1$			
	(4,2,1,3),(1,3,4,2),(2,4,3,1),(3,1,2,4)}	$\omega_{4}=4,n_{4}=1$			

## Abbreviations

The following abbreviations are used in this manuscript:

CE	Cluster Expansion
CVM	Cluster Variation Method
HEA	High-Entropy Alloy
*K*	Number of components in alloy
DFT	Density Functional Theory
GS	Ground States
G	Space Group of the disordered high temperature structure
$\left(\omega_{i},n_{i}\right)$	*i*-th sub-cluster of a maximal cluster (ω,n)
$N_{\omega_{i},n_{i}}$	operators g∈G acting on the sites of cluster $\left(\omega_{i},n_{i}\right)$ to rearrange the sites i.e., a permutation
$N_{\omega_{i},n_{i}}$	site multiplicity of the cluster $\omega_{i}$
$N_{\omega ,n}^{\beta }$	sub-cluster multiplicity of the cluster β∈(ω,n)
Ω[ω,n]	Number of times a cluster ω,n is contained in a supercell structure, generally used for Monte Carlo simulations, for obtaining thermodynamic quantities.
